# Adult Critical Care Electroencephalography Monitoring for Seizures: A Narrative Review

**DOI:** 10.3389/fneur.2022.951286

**Published:** 2022-07-15

**Authors:** Sonali Sharma, Michelle Nunes, Ayham Alkhachroum

**Affiliations:** ^1^Department of Neurology, University of Miami, Miami, FL, United States; ^2^Department of Neurology, Jackson Memorial Hospital, Miami, FL, United States

**Keywords:** EEG, critical care, seizures, monitoring, quantitative EEG

## Abstract

Electroencephalography (EEG) is an important and relatively inexpensive tool that allows intensivists to monitor cerebral activity of critically ill patients in real time. Seizure detection in patients with and without acute brain injury is the primary reason to obtain an EEG in the Intensive Care Unit (ICU). In response to the increased demand of EEG, advances in quantitative EEG (qEEG) created an approach to review large amounts of data instantly. Finally, rapid response EEG is now available to reduce the time to detect electrographic seizures in limited-resource settings. This review article provides a concise overview of the technical aspects of EEG monitoring for seizures, clinical indications for EEG, the various available modalities of EEG, common and challenging EEG patterns, and barriers to EEG monitoring in the ICU.

## Introduction

Electroencephalography (EEG) provides a continuous, non-invasive, and relatively inexpensive monitoring of cerebral function in real time that allows for an immediate detection of cerebral activity (1). Although seizure detection is the primary cause to obtain an EEG, several other important clinical indications have emerged over the years. Several guidelines from the Neurocritical Care Society (NCS), American Clinical Neurophysiology Society (ACNS), and European Society of Intensive Care Medicine (ESICM) are available to clinicians on the appropriate clinical scenarios that require EEG monitoring (2–4). The ACNS has recently updated a standardized set of critical care EEG terminology to assist with the identification and classification of clinically significant abnormal electrocerebral patterns (5).

Although the ideal application of continuous EEG (cEEG) is the standard 21-electrode montage from the International 10–20 system applied by a trained technician, the demanding critical care setting may not permit this in resource-limited areas (6, 7). Rapid application of limited EEG montages is now available to reduce not only the technician's time to apply EEG leads, but also to reduce the time to detect electrographic seizures (8–11). Furthermore, quantitative EEGs (qEEG) provides a computational analysis of the EEG signal that allows for the rapid review of large amounts of data accumulated over several hours in the intensive care unit (ICU) (12–22).

This review article provides a concise overview of seizure monitoring in the ICU, the technical aspects of EEG monitoring, clinical indications of EEG, the various modalities of EEG, common and challenging EEG patterns, and barriers to EEG monitoring.

## Methods

A PubMed/Medline literature search was performed for relevant articles published from January 2000 to May 2022, using the following search terms: “adult critical care EEG,” “adult neurocritical care EEG,” “continuous EEG,” and “quantitative EEG.” The search was limited to articles describing human subjects that were published in the English language. Clinical trials, meta-analysis, review articles, and practice guidelines were all eligible for inclusion. Abstracts were subsequently reviewed and included for relevance. Pertinent topics identified after full text review were also included when possible.

## Technical Aspects of EEG Monitoring in the ICU

The EEG is a differential amplifier - an apparatus that measures the voltage difference in electrical potential between two inputs while amplifying the difference (6). The electrical signal recorded by the EEG is generated by local field potentials from ionic currents flowing in the extracellular space by the pyramidal neurons in the cortical layers (6, 23). Synchronous activation of at least 10 cm^2^ of cortex is required to produce an electrical signal (24).

Scalp disk electrodes are the most common type of electrode used in EEG. However, subdermal needles and wire electrodes are also available (25). Disk electrodes are created by inert silver-silver chloride or gold metal held to the scalp by collodion to avoid interference with the electrical recording (25). The contact impendence should be between 1–10 kΩ and 5–10 kΩ typically accepted by most EEG laboratories (26). However, magnetic resonance imaging (MRI) and computed tomography (CT) compatible plastic electrodes are also available, and considered standard of care in the ICU (27–29).

Scalp electrodes should be arranged using the International 10–20 system with a 21-electrode montage. The abbreviations used on the EEG include: Fp (frontal-polar), F (frontal), C (central sulcus), P (parietal), O (occipital), T (temporal), and Z (sagittal) (6). Even numbered electrodes are located on the right hemisphere, while odd numbered electrodes are located on the left hemisphere (6). The lower integer electrodes are closer to the midline, while the larger integer electrodes are furthest away from midline (6). Additional channels may be added while in the ICU including a one-channel electrocardiogram, electromyography, respiratory sensors, and horizontal/vertical axis electrodes around the eyes (6).

The more commonly used montages are the bipolar montages (which include the longitudinal bipolar and transverse bipolar montage), referential montages, and common average montages (6). If the net polarity of the electrical signal is negative, there is an upward deflection on the EEG while the opposite is true of a net positive polarity (6). The EEG signal is composed of different frequency bands, the typical adult frequency bands include delta (1–3 Hz), theta (4–7 Hz), alpha (8–12 Hz), beta (13–30 Hz), and gamma (30–100 Hz) (30). A standard routine EEG (rEEG) recording should include at least 20 min, a short-term EEG includes 1–8 h, and a cEEG is 12–24 h or more (6, 25).

Intracortical depth electrodes are available to monitor limited cortical area but not commonly used in clinical practice (31, 32). These electrodes are placed in stuporous or comatose patients through cranial bolts at the bedside and have a similar safety profile to other intracranial monitoring probes (31). A retrospective study of 61 patients admitted for multimodal intracranial monitoring showed that of the 19 patients (31%) undergoing intracranial EEG, only one developed a complication related to invasive monitoring (malfunction or dislodgement of the device) (33). Depth EEG may be more sensitive than the scalp cEEG monitoring for detecting non-convulsive seizures, but the clinical and outcome relevance is not well-established (31). In a retrospective study of 14 patients undergoing concurrent scalp and intracranial EEG, 10 patients demonstrated electrographic seizures with the intracranial EEG. This contrasted with the detection on the scalp EEG where two patients showed intermittently correlated ictal activity, another two patients showed non-ictal appearing rhythmic delta activity, and six patients showed no concurrent ictal activity (34).

**Figure 1:**
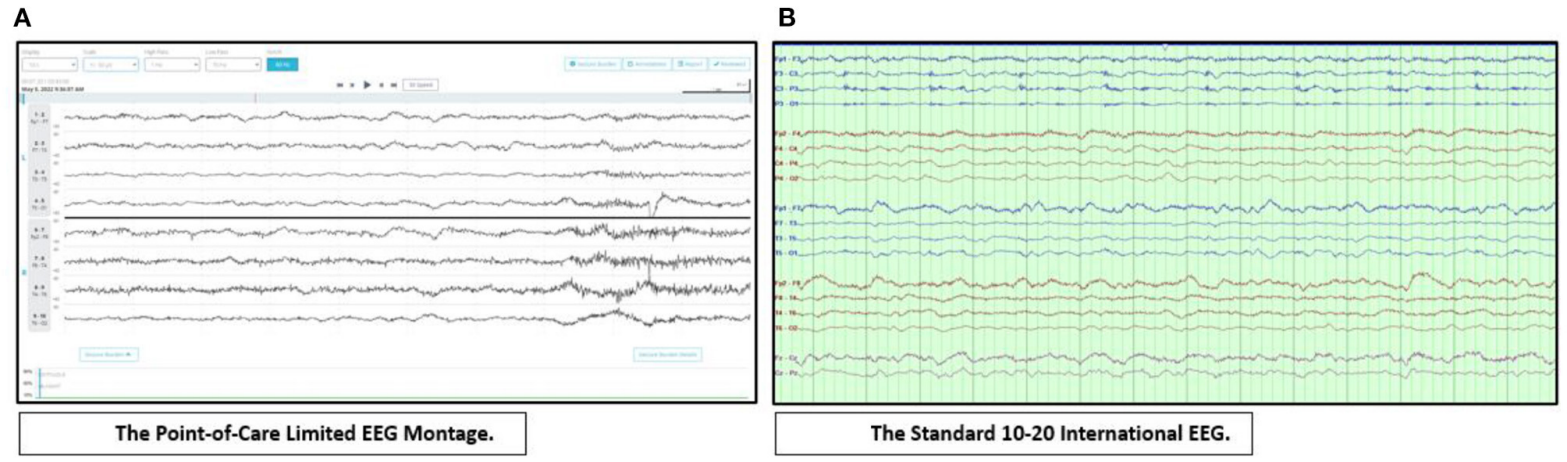
Point-of-care EEG. Suspected seizures in a 62-year-old man with decreased level of consciousness ruled out within 10 min with the use of a point-of-care limited EEG montage. The findings were later confirmed with the standard cEEG. **(A)** displays diffuse, irregular, attenuated mixed frequency delta-theta activity in the point-of-care limited EEG montage. Display: 10 s, Scale: ±50 μV, High Pass: 1 Hz, Low Pass: 70 Hz, Notch: 60 Hz. **(B)** displays diffuse, irregular, attenuated mixed frequency delta-theta activity in the standard International 10–20 EEG. Bipolar montage, LFF: 1 Hz, HFF: 70 Hz, Notch: 60 Hz, Sensitivity: 7 μV/mm, Timebase: 30 mm/s.

When issues of availability arise for the full 21-electrode EEG montage, limited montages may be used in the ICU setting. These include the sub-hairline (4 bipolar derivation—bilateral temporal and frontal) EEG montage and the FDA approved 8-channel rapid response headband EEG system (8–11). Figure 1 illustrates the utility of rapidly applying a point-of-care EEG in a critically ill patient.

A prospective study of 170 critically ill patients was conducted comparing the full-montage 10–20 placement of electrodes to the sub-hairline electrode montage. Out of the 8% of patients with seizures, the specificity to detect seizures with the sub-hairline montage was 100%, however, the sensitivity was only 54% (8). In another prospective study of 70 patients in a medical-surgical ICU who were simultaneously connected with a full 10–20 system and the four-channel sub-hairline montage demonstrated a sensitivity of 68% and specificity of 98% for seizure detection for both focal and generalized seizures (9).

Several important studies regarding the FDA approved eight-channel rapid response headband EEG system garnered confidence in its use in the ICU. In a small prospective study comparing 10 patients using the rapid response EEG device to the 20 patients using the 18-electrode EEG montage, the time to diagnosis of status epilepticus and on-call work force demand decreased. Mean time to interpretation was 23.8 min using rapid response EEG vs. 126.5 min when using the 18-channel (10). In a recent larger prospective multicenter non-randomized observational study (DECIDE trial) of five academic hospitals in the US, 164 critically ill patients were evaluated for possible non-convulsive status epilepticus (NCSE) by using the rapid response electroencephalography system. With the use of the device compared to clinical diagnosis alone, the sensitivity of the physician's electrographic seizure diagnosis improved from 77.8 to 100%, and the specificity improved from 63.9 to 89%. Time to EEG placement was a median of 5 min with the rapid response system vs. 239 min with conventional EEG (11). Furthermore, this rapid response modality may be economically feasible for both resource limited and rich regions but requires further investigation (35).

## Indications for EEG Monitoring in the ICU

The clinical use for EEG increased with the growing indications in critically ill patients. In a retrospective cross-sectional study with the National Inpatient Sample Data from 2004 to 2013—with more than 7,000,000 critically ill patients identified, of whom 22,728 received EEG—it was found that there was a >10-fold increase in EEG use from 0.06 to 0.8% by the end of the study (36). Despite this increase in EEG use, the EEG remains an underutilized tool. In a prospective multicenter observational study, only 37% of patients had EEG monitoring in those who met at least one of the indications for EEG monitoring (as per the ESICM) (37).

Several different critical care and neurophysiology societies provide guidelines for the indications of EEG in the ICU (2–4). The main recommendations include seizure detection for: (1) patients with convulsive status epilepticus (CSE) without return to baseline; (2) comatose patients with or without brain injury and without clear explanation of their mental status; and (3) unresponsive hypoxic-ischemic brain injury (HIBI) patients, during hypothermia, and within 24 h of rewarming (2–4). Other indications include the use of EEG to detect delayed cerebral ischemia in subarachnoid hemorrhage patients, prognostication after coma especially in patients with HIBI, and for monitoring of continuous sedation. It is important to note that the current 2020 American Heart Association adult post-cardiac arrest care algorithm recommends EEG in HIBI patients who are not following commands (38).

### Continuous EEG (cEEG) vs. Intermittent Routine EEG (rEEG)

Most seizures are non-convulsive in the ICU, making EEG a critical tool for the detection and management of ictal events (39, 40). In a systematic review and meta-analysis study of over 20,000 critically ill adult patients with concern for seizures, cEEG was superior to rEEG in detecting non-convulsive seizures (NCS) and non-convulsive status epilepticus (NCSE). The prevalence of detection for NCS, NCSE, and either NCS or NCSE by using cEEG was 17.9, 9.1, and 15.6%, respectively. The corresponding prevalence was high in post-CSE (33.5, 20.2, and 32.9%), central nervous system (CNS) infection (23.9, 18.1, and 23.9%), and post-cardiac arrest patients (20.0, 17.3, and 22.6%). This was in comparison to patients suffering from subarachnoid hemorrhage, intracerebral hemorrhage, subdural hemorrhage, acute ischemic stroke, sepsis, and traumatic brain injury (39).

A recent multicenter randomized clinical trial in Switzerland (CERTA study) studied 364 patients using either the cEEG (30–48 h total) or two rEEGs (20 min each). Continuous EEG was associated with increased detection of ictal and interictal features, however, the primary outcome of mortality at 6 months was similar between cEEG and rEEG (40). The study should reassure providers with limited-resource settings, but it does not support that cEEG should be abandoned when available (41).

To improve upon seizure risk stratification and the cost-effectiveness of cEEG, the 2HELPS2B score may be used. The 2HELPS2B score identifies the risk of seizures based on five EEG features (42, 43). The algorithm provides a total score of seven divided into points of 1 for EEG patterns with frequencies >2 Hz, one for independent sporadic epileptiform discharges, one for lateralized periodic discharges (LPD)/bilateral independent periodic discharges (BIPD)/lateralized rhythmic delta activity (LRDA), one for plus features (superimposed rhythmic, fast, sharp), one for a prior seizure, and finally two for brief potentially ictal rhythmic discharges (BIRD) (42, 43). In a multicenter retrospective analysis of 2,111 patients with a median cEEG duration of 48 h (total of 5,427 studies), the 2HELPS2B score was validated as a clinical tool to aid in seizure detection. The conclusion of this study was that a 1-h rEEG displaying no epileptiform discharges was an adequate screen to rule out electrographic seizures in critically ill patients who did not have a history of epilepsy. However, in patients with highly epileptiform EEG patterns during the first hour (2HELPS2B score of ≥2) a cEEG of at least 24 h was recommended (42, 43).

### Quantitative EEG (qEEG) in the ICU

The real-time visual analysis and interpretation of the raw EEG possesses multiple challenges. First, there may not be a 24-h availability of an experienced electroencephalographer for real-time interpretation of the data. Second, the sheer volume required to interpret the data takes significant time and effort. Third, subtle changes to trends on raw EEG may be missed by even the most experienced electroencephalographer (12). Quantitative EEG (qEEG) mitigates this burden by allowing the rapid review of a large volume of EEG data in a simplified display (12, 13).

The mathematical algorithms utilized in qEEG is beyond the scope of this review. In brief, the EEG signal is a collection of sinusoidal waves with key properties utilized by the mathematical algorithms to produce different qEEG panels or trends (14). The color spectrogram power scale is measured in decibels (dB) with cooler colors representing lower power and warmer colors representing higher power. Seizures are most easily recognized on a spectrogram by a “flame” appearing pattern due to the abrupt increase in power across a range of frequencies that stands out from the background (14).

The commonly used qEEG panels or trends include compressed spectral array (CSA), density spectral array (DSA), asymmetry relative spectrogram, the fast Fourier transform (FFT) spectrogram, the rhythmicity spectrogram, the amplitude EEG (aEEG), and the seizure detector panel. The compressed spectral array (CSA) generates a three-dimensional display by plotting successive epochs as a function of time (12). The density spectral array (DSA) depicts EEG spectral power amplitude as a gray-scale or color intensity function rather than vertical deflections as seen in the CSA (12). The asymmetry relative spectrogram displays power differences between homologous electrodes at discrete frequencies and illustrates power asymmetry across the two hemispheres (15). It is a line graph that displays an average of the absolute values over a specified frequency range or relative asymmetry data as a function of time (15). The fast Fourier transform (FFT) spectrogram displays color coded power of EEG at different frequencies using a fast Fourier transform analysis of the amplitude of waveforms as a function of time (15). The rhythmicity spectrogram displays a three-dimensional representation of the power characteristics for the EEG and a density spectral array of frequencies as a function of time. It is a graphical depiction of the amplitude of primary rhythmic EEG components present in four frequency bands: 1–4, 4–9, 9–16, and 16–25 Hz (15). Amplitude EEG (aEEG) spectrogram displays amplitude characteristics of the EEG as a function of time (15). The seizure detector trend displays the combination of multiple inputs as a seizure probability, dichotomized into a value of zero or one (15).

**Figure 2:**
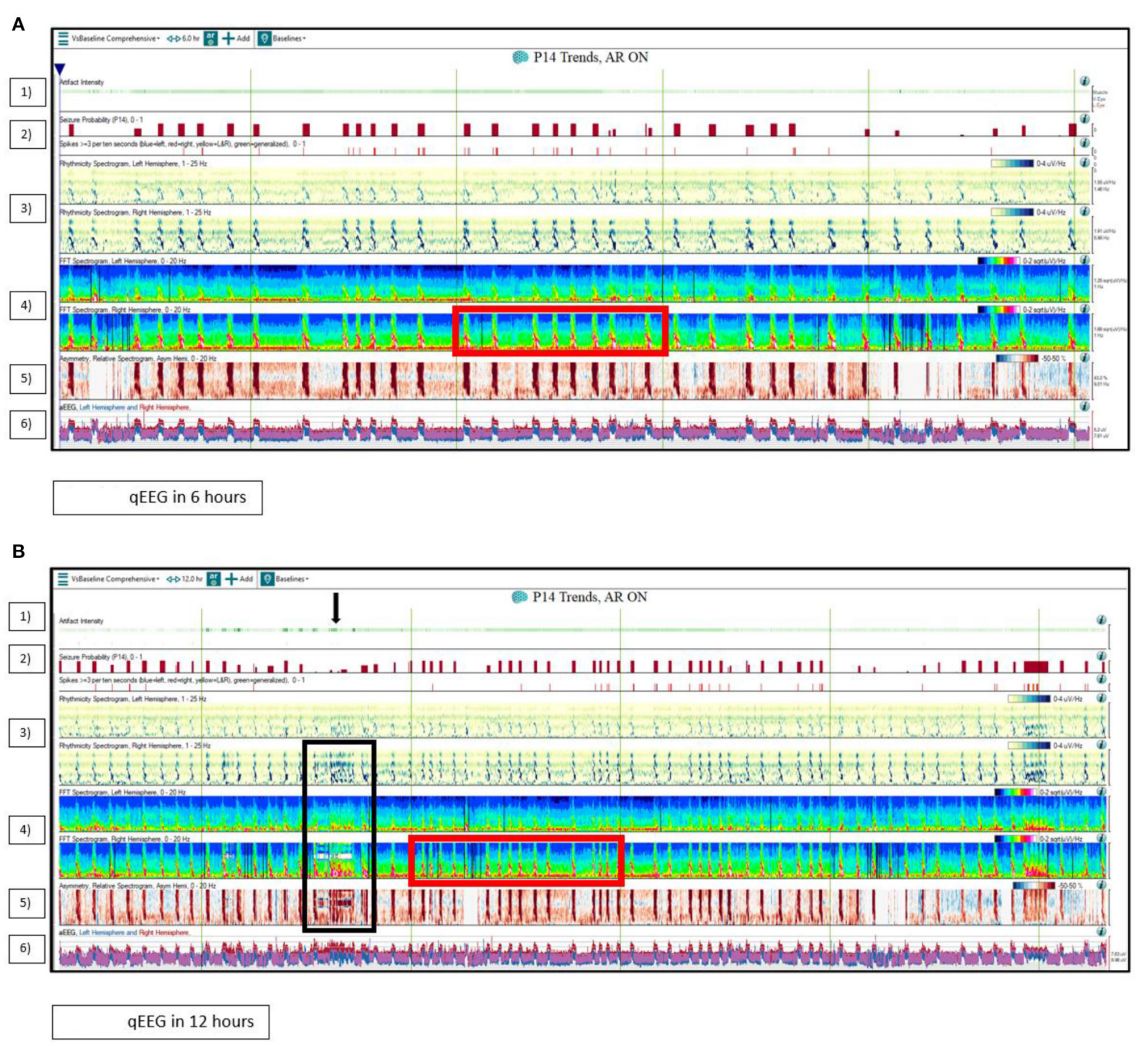
Quantitative EEG (qEEG) use in the ICU. The quantitative EEG (qEEG) in a 71-year-old man with presumed autoimmune/paraneoplastic encephalitis with focal right temporal electro-clinical status epilepticus. **(A)** displays the qEEG panels in a 6-h window, while **(B)** displays the qEEG panels in 12 h. (1) The artifact intensity panel displays the likelihood that an abrupt change seen on other qEEG trends is likely due to an artifact. Note the abrupt abnormality due to EEG lead disruption in **(B)** on the right hemisphere rhythmicity spectrogram, the right hemisphere FFT spectrogram, and the relative asymmetry spectrogram that was detected by the artifact intensity panel (Black arrow and black box). (2) The seizure probability (0–1) panel provides a value between 0 and 1 to indicate the seizure probability as estimated by the software. (3) The left and right rhythmicity spectrogram panel displays the power characteristics for each frequency band (1–4, 4–9, 9–16, and 16–25 Hz) as a function of time. Note the increased power peaks over the right rhythmicity spectrogram panel. (4) The left and right FFT (fast Fourier transform) spectrogram panel displays the color-coded power of different EEG frequency bands as a function of time. Note the distinct “flame” appearing peaks in the right FFT spectrogram panel indicating discrete right temporal seizures (Red box). (5) The relative asymmetry spectrogram panel (left in blue and right in red) displays power differences between homologous electrodes at discrete frequencies and illustrates power asymmetry across the two hemispheres. Note the several peaks in red. (6) The left (in blue) and right (in red) hemisphere amplitude EEG (aEEG) panel displays amplitude characteristics of the EEG as a function of time. Note the higher amplitudes over the right hemisphere.

In an ICU qEEG Survey conducted in 2016, 75 neurophysiologists from the ACNS responded that they utilized qEEG for seizure detection (92%), burst suppression monitoring (58.7%), and prognosis for cardiac arrest (21.3%). The most frequently used qEEG trends or panels for seizure detection were rhythmicity spectrogram (61%), automated seizure detector (55%), color density spectral array (CDSA)/compressed spectral array (CSA)/density spectral array (DSA)/fast Fourier transformation (FFT) spectrogram (47%), asymmetry index/asymmetry spectrogram (43%), and amplitude-integrated EEG (aEEG) (41%) (13). Figure 2 illustrates a case on the utility of qEEG in a critically ill patient suffering from focal electro-clinical status epilepticus.

Several studies assessed the diagnostic accuracy of qEEG for seizure detection in critically ill patients. One of the earliest key studies was conducted by Stewart et al., which demonstrated acceptable sensitivity and false-positive rates of CDSA and aEEG for seizure detection in critically ill patients (44). In a cohort of 562 seizures from 58 pediatric and adult patients, the overall sensitivity of the qEEG spectrograms for detecting seizures ranged from 43 to 72%. The highest sensitivity (402/562, 72%) was detected by the seizure detection trend. The asymmetry spectrogram had the highest sensitivity for detecting focal seizures (117/125, 94%). The FFT spectrogram was most sensitive for detecting secondarily generalized seizures (158/187, 84%). Finally, the seizure detection trend was the most sensitive for generalized onset seizures (197/250, 79%) (15). In one retrospective study of 118 adult patients, the CSA-guided review vs. the gold standard visual analysis of the raw EEG was sensitive for seizure detection at 87.3%, periodic epileptiform discharges at 100%, rhythmic delta activity at 97.1%, focal slowing at 98.7%, generalized slowing at 100%, and epileptiform discharges at 88.5%. The average time to review 24 h of cEEG data was 8 (±4) min for CSA-guided review and 38 (±17) min for visual analysis of the raw cEEG (16). In a second retrospective study of 118 critically ill adult patients, the overall detection rate of CSA-guided review of cEEG for seizures was 89.0% of 1,190 total seizures, 94% for epileptiform discharges, 100% for periodic epileptiform discharges, rhythmic delta activity, and both focal and generalized slowing (17). In a study of 6-h EEG epochs from 15 critically ill adult patients undergoing qEEG compared with the gold standard of the neurophysiologist analyzing raw EEG, the mean sensitivity for seizure identification ranged from 51 to 67% for qEEG-only read and 63 to 68% for qEEG and raw EEG analysis together. The false-positive rates for qEEG-only read was 1/h and 0.5/h for both qEEG and raw EEG analysis. The median time for review was shorter for qEEG (6 min) and qEEG plus raw EEG review (14.5 min) compared to only raw EEG review (19 min) (19).

Finally, qEEG may also be a useful tool for the non-electroencephalographers. In a prospective, single-institution study of neurocritical care nurses' ability to detect seizures on qEEG, the false positive rate was 0.1/h (20). In another prospective single-institution study of 109 critically ill adult patients, the neurocritical care nurses' sensitivity and specificity of detecting seizures from qEEG panels (rhythmicity spectrogram and aEEG) at bedside was 74 and 92%, respectively (21). A retrospective study analyzed the sensitivity and specificity of neurophysiologists and non-neurophysiologists' ability to detect seizures on qEEG in 45 ICU patients with 1-h qEEG panels (180 studies). The data showed the sensitivity and specificity was 87 and 61% for neurophysiologists, 80 and 80% for EEG technologists, and 87 and 61% for neurocritical care nurses, respectively (18). Another retrospective study evaluated the accuracy of seizure burden in 69 critically ill adult patients with super-refractory status epilepticus by using qEEG reviewed by three sets of reviewers: (1) Two board-certified neurophysiologists using raw EEG (gold standard), (2) Two neurocritical care providers with substantial qEEG analysis expertise (qEEG experts), and (3) Two inexperienced qEEG readers (qEEG novice). The raw EEG experts identified 2,950 total seizures in 25 patients, the qEEG experts had 93% sensitivity, 61% specificity, a false positive rate of 6.5 per day, and good agreement (*k* = 0.64) between both qEEG experts, and the qEEG novices had 98.5% sensitivity, 13% specificity, a false positive rate of 15 per day, and fair agreement (*k* = 0.4) between both qEEG novices (45).

## Common ICU EEG Patterns

### Electrographic Seizure, Electroclinical Seizure, and Status Epilepticus

Electroclinical seizures are defined as paroxysmal events during which a clinical change is accompanied by a characteristic abnormal EEG pattern. The typical changes seen on EEG are, as defined by the ACNS and other societies (5).

1) Repetitive spikes, sharp waves, spike/sharp waves, and slow-wave complexes with a frequency >3 Hz.
2) Repetitive rhythmic waves with either an incrementing onset, decrementing offset, and/or post-discharge slowing or attenuation.
3) Repetitive spikes, sharp waves, spike/sharp waves, and slow-wave complexes with a frequency of 3 Hz or less, and significant improvement in clinical state or EEG background after administration of ASMs (5, 46).

**Figure 3:**
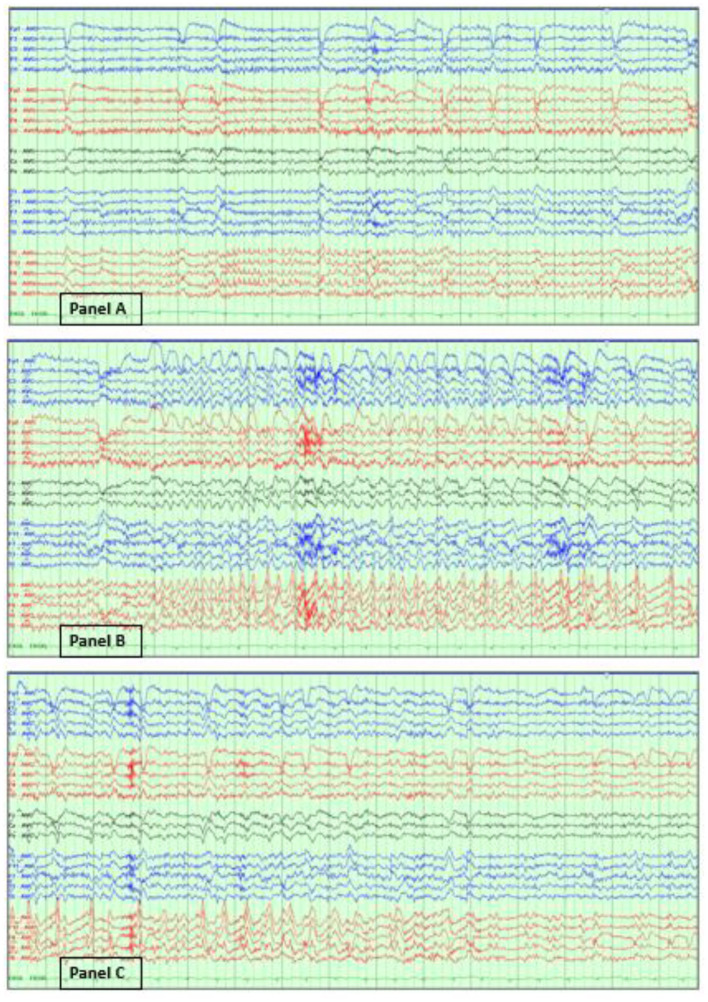
Unequivocal electrographic seizure on EEG in the ICU. Right temporal electrographic seizure **(A–C)** in a 21-year-old woman with herpes simplex (HSV) encephalitis. Average referential montage with double density electrodes added in the bilateral basal temporal regions (T1, F11, T2, F12), LFF: 1 Hz, HFF: 70 Hz, Notch: 60 Hz, Sensitivity: 10 μV/mm, Timebase: 30 mm/s.

In contrast, electrographic seizures in the absence of a clear clinical change are more challenging to determine. The 2021 ACNS terminology, which is briefly explained below, excludes the unequivocal electrographic seizure definition, which is defined as generalized spike-waves at 3 Hz or more, and/or evolving discharges that reach >4 Hz (5, 46). Figure 3 illustrates an example of an unequivocal electrographic seizure.

The diagnosis of NCSE relies exclusively on EEG interpretation. NCSE is a condition with high morbidity and mortality and its prompt diagnosis by the clinician is paramount to the care of the critically ill patients (47). Although several criteria have been proposed, the Salzburg criteria has been widely accepted (48). Table 1 describes the criteria in further details.

**Table 1:** The Salzburg criteria for the diagnosis of NCSE (48).

One of the following criteria must be met and be continuously present for 10 s or more for NCS and 30 min for NCSE:
i. Epileptiform patterns occurring >2.5 Hz
ii. Concurrent subtle clinical accompaniments
iii. Spatiotemporal evolution

The most updated 2021 ACNS terminology was designed to standardize the terminology of periodic and rhythmic EEG patterns in critically ill patients. Its basic premise consists of a main term #1 followed by a main term #2, with modifiers added if appropriate. Main terms #1 refer to localization and include: generalized, lateralized, bilateral independent, and multifocal. Main terms #2 is the description of the activity seen and include: periodic discharges (PDs), rhythmic delta activity (RDA), and spike-and-wave or sharp-and-wave (SW). Modifiers include prevalence, duration, frequency, number of phases, sharpness, amplitude, polarity, stimulus induced, evolving or fluctuating, and plus (+) features, -with the latter describing features of a more ictal appearing pattern (5).

### Periodic Discharges (PDs)

Periodic discharges (PDs) are classified as discharges with uniform morphology and duration with a clear inter-discharge interval between consecutive waveforms, and recurrence of the waveform at nearly regular intervals (5). Periodic discharges are subclassified according to their location described as either generalized, lateralized, regional, or bilateral with a variety of prognoses.

#### Generalized Periodic Discharges (GPDs)

Generalized periodic discharges (GPDs) are bilaterally synchronous and symmetric, however, they can be frontally, occipitally, or midline predominant (5). GPDs are associated with NCS and NCSE. However, this association is less common when compared to lateralized (LPDs) and bilateral independent periodic discharges (BIPD) (49). Furthermore, the most common etiologies associated with GPDs are often seen in acute brain injury, ischemic/hemorrhagic stroke, and hypoxic-ischemic brain injury (HIBI) patients (50).

**Figure 4:**
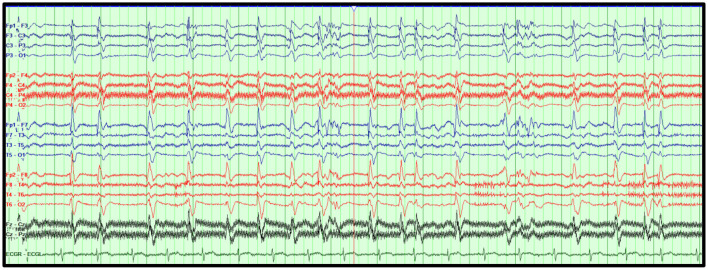
Generalized periodic discharges (GPDs). GPDs at 1–2 Hz superimposed on a diffusely attenuated delta background in an 82-year-old woman who suffered HIBI. Bipolar montage, LFF: 1 Hz, HFF: 70 Hz, Notch: 60 Hz, Sensitivity: 7 μV/mm, Timebase: 30 mm/s.

The morphology of the GPDs may also portend to a specific clinical diagnosis and/or outcome. For example, GPDs with a triphasic morphology, also known as triphasic waves, have long been associated with toxic-metabolic etiologies (51–53). There is evidence, however, that this association may not be accurate, and that GPDs with triphasic morphology maybe associated with developing seizures (54, 55). Features associated with poor outcomes have been described with static GPDs, superimposed faster frequencies, lack of triphasic morphology, and anterior to posterior phase lag (55). Figure 4 illustrates a case of GPDs in a critically ill HIBI patient associated with poor outcome.

#### Lateralized Periodic Discharges (LPDs)

**Figure 5:**
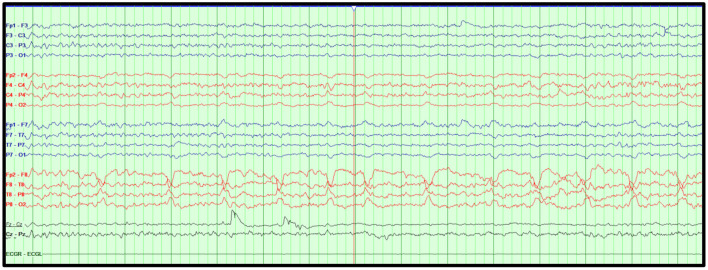
Lateralized periodic discharges. Right temporal periodic discharges in an 18-year-old man with temporal lobe epilepsy with medication non-adherence. Bipolar montage, LFF: 1 Hz, HFF: 70 Hz, Notch: 60 Hz, Sensitivity: 7 μV/mm, Timebase: 30 mm/s.

Lateralized periodic discharges (LPDs) are asymmetric discharges that are either unilateral or bilateral (5). This pattern was previously called periodic lateralized *epileptiform* discharges (PLEDS) but given the controversy of whether a specific LPD pattern is or is not epileptic, the epileptiform portion was removed from the ACNS guidelines in 2012 (56). LPDs are the most common periodic pattern in the ICU seen in up to 6–9% of hospitalized patients (57). LPDs may occur in the setting of ischemic or hemorrhagic stroke, traumatic brain injury, encephalitis, epilepsy, systemic infections, and other toxic/metabolic related etiologies. LPDs are highly associated with seizures (40–60%) (51, 57, 58). A study conducted in 2017 found that LPDs at frequencies higher than 2 Hz cause cerebral metabolic decompensation with an increase in regional cerebral blood flow and decrease in brain oxygenation indicating tissue hypoxia, which resembles the physiological changes seen in seizures (59). It can manifest as an electrographic pattern only or clinically as focal seizures, generalized seizures, or epilepsia partialis continua (59). Figure 5 illustrates a case of right temporal LPDs.

### Lateralized/Generalized Rhythmic Delta Activity (LRDA/GRDA)

Rhythmic delta activity refers to a repetition of a waveform with relative uniform morphology and no interval between consecutive waves (5). The terms lateralized and generalized follow the same rules as periodic discharges.

Lateralized rhythmic delta activity (LRDA) is highly associated with seizures, at an incidence similar to LPDs, with the risk increasing in the presence of any Plus modifiers (49, 60). On the contrary, generalized rhythmic delta activity (GRDA), previously referred to as FIRDA (frontal intermittent rhythmic delta) and OIRDA (occipital intermittent rhythmic delta), is not associated with an increased risk of seizures, regardless if Plus modifiers are present (60).

### Burst Suppression/Attenuation Pattern

A burst-suppression pattern is an EEG pattern characterized by a quasi-periodic high amplitude “burst” alternating with periods of suppression (<10 μV) or attenuation (≥10 μV but <50% of the highest voltage background) (5). This EEG pattern can be physiologic (early development in the pre-mature brain), or pathological as seen in HIBI and severe epileptic encephalopathies of infancy (61–63). It can also be induced by anesthetics or hypothermia, which are commonly used to treat status epilepticus and uncontrolled elevated intracranial pressure in patients suffering from brain injury (64, 65).

A burst suppression/attenuation pattern identified in HIBI is typically associated with a poor prognosis (63, 66). For example, the presence of burst suppression with identical bursts had 100% specificity for a poor prognosis (66). This pattern is also highly associated with seizure recurrence (67).

## Challenging ICU EEG Patterns

### Ictal-Interictal Continuum (IIC)

The concept of IIC was first introduced by Pohlmann-Eden et al. (58) The authors initially described LPDs as an active state in which “unstable neurobiological processes create an ictal-interictal continuum, with the nature of the underlying neuronal injury, the patient's pre-existing propensity to have seizures, and the co-existence of any acute metabolic derangements all contributing to whether seizures occur or not” (58). At the time of this review, the ACNS recognizes that this is still a term under development without broad consensus, yet, potentially ictal and often warrants a diagnostic treatment trial. Currently, IIC includes rhythmic and periodic patterns occurring at 1–2.5 Hz without spatial evolution or clinical correlate (5). The increased risk for seizures associated with IIC patterns has been well-established, particularly with frequencies >1.5 Hz (49). However, it is still unclear whether IIC causes a similar degree of neuronal injury, worsening outcomes, or require the same degree of aggressive anti-seizure medication (ASM) treatment as do the definitive electrographic seizures.

### Generalized Periodic Patterns With Triphasic Morphology

**Figure 6:**
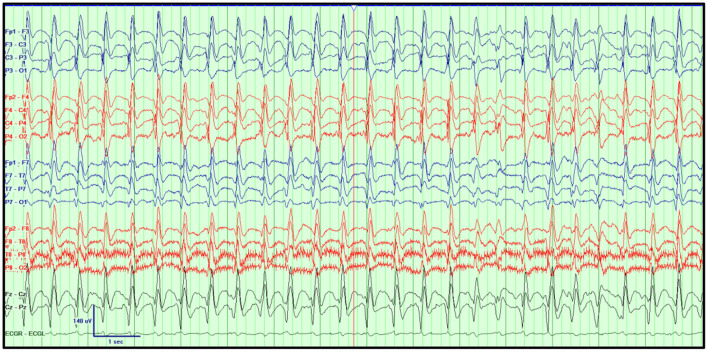
Generalized periodic pattern with triphasic morphology. GPDs with a triphasic morphology at 1–2 Hz with a posterior to anterior gradient superimposed over a diffuse, irregular, delta-theta background in a 52-year-old woman with altered mental status, chronic kidney disease and sepsis treated with cefepime. Bipolar montage, LFF: 1 Hz, HFF: 70 Hz, Notch: 60 Hz, Sensitivity: 7 μV/mm, Timebase: 30 mm/s.

The GPDs with triphasic wave morphology, commonly known as triphasic waves (TWs), was first described by Brickford and Butt (68). Triphasic waves consist of (1) three phases with an initial fast and low-amplitude negative phase, followed by a second positive phase, and finally a third high-amplitude negative phase; (2) occur usually at a frequency of 1.5–2 Hz; (3) has an anterior to posterior, or posterior to anterior lag; and (4) a bi-frontal predominance (69). It was initially associated with hepatic encephalopathy; however, it has since been associated with a variety of metabolic, structural, and toxic encephalopathies. These include, but are not limited to, uremia, hypoglycemia, hyperthyroidism, sepsis, toxicities from various drugs (cefepime, baclofen, and valproic acid), vascular disease, and dementia (51, 70, 71). Triphasic waves may assume an ictal pattern and be associated with seizures. This is particularly true in high-risk patients with a focality on EEG, inter-burst suppression, a history of epilepsy, and abnormal neuroimaging findings (72). Various authors have suggested an algorithm to distinguish ictal appearing TW pattern to facilitate correct therapeutic intervention and avoid unnecessary use of ASMs (69). Figure 6 illustrates a patient with toxic-metabolic encephalopathy that was confirmed with cEEG.

### Stimulus Induced/Terminating Patterns

Stimulus induced rhythmic, periodic, or ictal discharges (SIRPIDs) was described initially in 2004 by Hirsch et al. (73). It is encountered in roughly 10–22% of patients undergoing EEG in the ICU and consist of any rhythmic, periodic, or ictal discharge induced by an alerting stimulus, such as noise, sternal rub, physical examination, suctioning, turning, and other activities related to patient care (73, 74). SIRPIDs include stimulus induced (SI)- periodic discharges, rhythmic delta activity, seizures, and IIC.

A multicenter, international, retrospective study found that SIRPIDs are not associated with worsening mortality after the data was adjusted for other prognostic factors such as age, anoxic brain injury, and absent reactivity on EEG (74). Similar findings were published in the literature (75, 76). SIRPIDs are also suggested to be associated with good prognosis in comatose survivors after cardiac arrest (77). Anti-seizure medications are commonly used to treat SIRPIDs, however, its clinical utility remains uncertain.

### EEG Artifacts/Seizure Mimics

Unfortunately, particularly in the ICU setting, several artifacts may obscure the EEG signal. It is imperative for the electroencephalographer to properly identify and attempt to mitigate these artifacts to avoid the misdiagnosis of “noise” as cerebral electrographic abnormalities. This “noise” is detected by EEG electrodes from varying sources contaminating the EEG signal. It is not infrequent for artifact to disrupt the EEG background, obscuring underlying electrographic abnormalities, potentially obscuring electrographic seizures, and/or mimicking ictal patterns. The most common sources of EEG artifact in the ICU are related to (1) physiologic features such as sweat, eye flutter, movements, nystagmus, cardiac cycle, pulse, chest compression, and ventilator-related artifacts; (2) instrument and electrode artifact such as the 50 or 60 Hz electrical artifact; and (3) artifacts from multiple electronic devices (i.e., feeding machines) (78).

## Common Seizure Etiologies in the ICU

### Seizures in Patients With Impaired Level of Consciousness

Non-convulsive status epilepticus is an underrecognized cause of impaired level of consciousness in the ICU, particularly in septic patients. In a prospective study of 236 critically ill comatose patients with no clinical signs of seizure, 8% were found to have NCSE with EEG evaluation (79).

In a retrospective study of 154 adult surgical ICU patients who underwent cEEG for altered mental status over a 6-year period, 16% of patients all suffering from sepsis developed NCS with 5% (*n* = 8) developing NCSE. Clinical seizures prior to cEEG were more common among comatose patients who developed NCS or NCSE compared to patients without clinical seizures (70 vs. 27%) (80).

In a retrospective study of 201 patients admitted to a medical ICU without a known acute neurological injury who underwent cEEG, 10% of patients developed electrographic seizures with the majority of septic patients developing electrographic seizures when compared to non-septic patients (32 vs. 9%) (81).

### Seizures in Post-convulsive Status Epilepticus (CSE)

As mentioned earlier, patients with convulsive seizures are at greater risk for non-convulsive status epilepticus. In a prospective study of 164 critically ill patients with CSE with clinical control of the seizures within 24 h, 48% of those patients continued to have persistent electrographic seizures with more than 14% meeting criteria for NCSE (82).

### Seizures in Traumatic Brain Injury

Traumatic brain injury also poses a risk for seizures in our critically ill patients. In a prospective study of 70 traumatic brain injury patients requiring intensive care, 33% developed seizures 74 h after the initial trauma (83). In another prospective study of 94 critically ill patients undergoing cEEG who suffered from moderate-to-severe traumatic brain injury, 21 (22%) of patients developed convulsive/non-convulsive seizures with six patients developing status epilepticus. In 52% of those patients, the seizures were NCS (84).

### Seizures in Subarachnoid Hemorrhage

Seizures developing in the aftermath of a subarachnoid hemorrhage are common in the ICU. In a prospective study of 101 patients with subarachnoid hemorrhage who survived the first 48 h of hospitalization, 26 of those patients were monitored with cEEG. Eight of those patients (8%) were diagnosed with NCSE with an average of 18 days after the subarachnoid bleed day. Risk factors for NCSE included a Hunt and Hess grade IV or V, older age, ventricular drainage, and cerebral edema on CT scans (85).

In another retrospective study of 11 out of 389 critically ill patients suffering from NCSE in the setting of spontaneous subarachnoid hemorrhage, the most common risk factors among the patients included advanced age, female sex, need for ventriculostomy, poor neurological grade (Hunt and Hess Grade III-V), thick cisternal blood clots, and structural lesions (intracerebral hemorrhage and stroke) (86).

### Seizures in Intracerebral Hemorrhage

Stroke, particularly hemorrhagic stroke, is a risk for seizures in the ICU. In a retrospective study of 102 patients with intracerebral hemorrhage who underwent cEEG, seizures occurred in 31% (*n* = 32) of patients with 18% (*n* = 18) of those patients developing electrographic seizures only. The first seizure was detected within the first 1 h of cEEG in 56% of patients and within 48 h in 94% of patients. Risk factors associated with seizures included an ICH volume of 30% or more between admission and the 24-h follow-up CT scan (87).

In a prospective study of 109 patients with 63 patients suffering from intraparenchymal hemorrhage (*n* = 63) undergoing cEEG, electrographic seizures occurred in 18 of 63 patients (28%) during the initial 72 h of EEG monitoring with most seizures occurring in lobar hemorrhages and 21% in subcortical hemorrhages (88).

Not only are acute seizures common in intracerebral hemorrhages, but late seizures—that is seizures occurring 7 days after hemorrhagic insult—may also occur. The CAVE score is designed to assist the intensivist with identifying patients most susceptible for late seizures after an intracerebral hemorrhage. The CAVE score (0–4 points) assigns points for the cortical involvement of intracerebral hemorrhage (1 point), patient age <65 years (1 point), hemorrhagic volume >10 ml (1 point), and early seizures within 7 days of the hemorrhagic insult (1 point). In a large retrospective study of 1,318 patients suffering from intracerebral hemorrhage, it was found that the risk for late seizures was 0.6, 2.6, 9.8, 34.8, and 46.2% for CAVE scores of 0–4, respectively (89).

### Seizures in Ischemic Stroke

Although seizures are more commonly seen in hemorrhagic strokes, it can also manifest in the setting of acute ischemic strokes. In a prospective study of 109 patients with 46 patients suffering from ischemic stroke undergoing cEEG, electrographic seizures occurred in 3 of the 46 patients (6%) during the initial 72 h of EEG monitoring (88).

In another prospective study of 100 adult patients undergoing cEEG with an acute ischemic (91 patients) and hemorrhagic stroke (9 patients), two patients with ischemic strokes developed focal electrographic seizures (90).

### Seizures in CNS Infection

Central nervous system infections—whether viral, bacterial, or fungal—are also common causes of seizures in the ICU. In a retrospective cohort study of 42 patients with a primary central nervous system infection—viral in 27 patients (64%), bacterial in eight patients (18%), and fungal/parasitic in seven patients (17%)—electrographic seizures were captured in 14 patients (33%) with only five of those patients developing a clinical correlate (91). In a prospective study of 62 critically ill adult patients with acute community acquired bacterial meningitis, 8 (12.5%) of the patients developed seizures (92).

An observational cross-sectional study of 696 episodes of community acquired bacterial meningitis in patients older than 16 years of age with confirmed CSF culture, seizures occurred in 121 patients (17%). The median time was 24 h between the first seizure and admission. Seizures were most common in patients with Streptococcus pneumonia, focal cerebral abnormalities, and a low Glasgow Coma Scale (93).

### Seizures in Hypoxic-Ischemic Brain Injury (HIBI)

As described earlier in this article, HIBI is a common cause for seizures in the ICU. In a retrospective observational study of 166 post-anoxic comatose patients admitted to an ICU (all but four patients with out-of-hospital arrest), 107 patients underwent cEEG. Out of the 107, 35 (33%) patients had post-anoxic status epilepticus (94). In a prospective study of 101 critically ill adult comatose post-cardiac-arrest patients who underwent cEEG, 12 (12%) of the patients suffered from NCSE with four patients experiencing NCSE within 8 h of cEEG recording and within 12 h after resuscitation from cardiac arrest (95).

In another prospective study of 103 out of 192 adult patients with cardiac arrest, predominantly out-of-hospital (*n* = 148 or 77%, compared to in-hospital *n* = 44 or 23%), undergoing hypothermia protocol, six patients developed status epilepticus when EEG was obtained on day 2 and 3 of initial injury (96). Finally, in a prospective observational study of 95 patients after cardiac arrest treated with hypothermia, 26 patients (27%) developed electrographic status epilepticus (97).

Since the prevalence of seizures is high in HIBI patients, a recent multicenter clinical trial known as the treatment of electroencephalographic status epilepticus after cardiopulmonary resuscitation trail (TELSTAR) was conducted to determine the degree of treatment required by this unique cohort (50, 98). This was a multicenter clinical trial that randomized open-label treatment assignments and blinded end-point evaluation of 172 adult post-cardiac arrest patients in 11 ICUs in Europe. The goal was to suppress rhythmic and periodic EEG patterns (including GPDs, electrographic seizures, evolving patterns) for at least 48 h along with standard ASM and targeted temperature management or to only treat with standard ASMs and targeted temperature management. The primary outcome was the neurological outcomes according to the Cerebral Performance Category at 3 months—a good outcome (absent to mild-moderate disability) to poor outcomes (severe disability, coma, or death). It was found that at 3 months, 79 of 88 (90%) in the treatment group and 77 of 84 (92%) in the control group had poor outcomes (*P* = 0.68). Mortality at 3 months was 80% in the treatment group and 82% in the control group. The authors highlight limitations to their study that included the trial physicians' ability to withdraw life-sustaining treatment after the 48-h treatment period, the study treating several different patterns that are not clearly ictal in nature, and finally the difficulty in evaluating a sick population who had poor outcomes at the onset (50, 98).

## Challenges and Future Directions

The benefits of utilizing cEEG in the ICU comes with its challenges (99). The surge of cEEG may add a burden among EEG technologist and electroencephalographers to cover the clinical need—a relevant issue in the American healthcare system (100, 101). Additionally, the access to cEEG monitoring is also challenging in resource-limited regions. Typically, serial rEEGs in lieu of cEEG are used in these regions. However, with the advent of remotely analyzed point-of-care EEGs, the financial burden may be alleviated in these settings (11, 40).

Further research is required to determine how aggressively challenging EEG patterns (such as IIC) should be treated, the appropriate seizure control or suppression ratio in status epilepticus, and patterns with triphasic morphologies.

The future of critical care EEG appears promising with the improving storage capacity, and the processing power allowing for machine learning utilization. This utilization is a useful tool for predicting seizures, and for an automated interpretation of large data sets (102, 103). However, these applications are not widely used in clinical practice and may not improve the workload of the electroencephalographers (104, 105).

## Conclusion

In conclusion, the EEG is an essential apparatus in critical care that provides a relatively inexpensive tool for clinicians to monitor cerebral activity in real time. Although the awareness of subtle electro-clinical and electrographic non-convulsive seizures has increased in critical care, cEEG continues to be underutilized. With the rise in cEEG monitoring, the burden falls to the electroencephalographer and the institution to provide this necessary instrument to our critically ill patients. However, with the introduction of qEEG and other future machine learning applications, we may find more efficient and less taxing means of acquiring this necessary electrocerebral data.

## Author Contributions

SS and MN wrote the manuscript. AA reviewed and finalized the manuscript, and made critical revisions. All authors contributed to the article and approved the submitted version.

## Funding

AA was supported by an institutional KL2 Career Development Award from the Miami CTSI NCATS UL1TR002736 and by the National Institute of Neurological Disorders, and Stroke of the National Institutes of Health under Award Number K23NS126577.

## Conflict of Interest

The authors declare that the research was conducted in the absence of any commercial or financial relationships that could be construed as a potential conflict of interest.

## Publisher's Note

All claims expressed in this article are solely those of the authors and do not necessarily represent those of their affiliated organizations, or those of the publisher, the editors and the reviewers. Any product that may be evaluated in this article, or claim that may be made by its manufacturer, is not guaranteed or endorsed by the publisher.
